# An effective strategy with tofacitinib for the management of focal myositis with rheumatoid arthritis: a case report

**DOI:** 10.3389/fimmu.2024.1502410

**Published:** 2024-12-24

**Authors:** Jing Pan, Haihong Zheng, Juan Xu, Shaobiao Pan

**Affiliations:** ^1^ Department of Rheumatology and Immunology, Taizhou Hospital of Zhejiang Province Affiliated to Wenzhou Medical University, Linhai, China; ^2^ Department of Interventional Centers, Taizhou Hospital of Zhejiang Province Affiliated to Wenzhou Medical University, Linhai, China; ^3^ Department of Pathology, Taizhou Hospital of Zhejiang Province Affiliated to Wenzhou Medical University, Linhai, China

**Keywords:** focal myositis, rheumatoid arthritis, tofacitinib, misdiagnose, case report

## Abstract

Focal myositis is a rare, localized, benign, self-limiting, and non-suppurative inflammatory lesion of the skeletal muscle that may occasionally occur as a complication of rheumatic diseases. This case report discusses a 58-year-old patient with rheumatoid arthritis, who was diagnosed with focal myositis during standard immunosuppressive therapy. The patient was treated with tofacitinib; to our knowledge, this is the first reported case of focal myositis managed with this medication. Clinicians should maintain a suspicion for focal myositis in patients presenting with localized muscle swelling and pain, especially those with underlying rheumatic conditions. In cases where corticosteroid tapering proves difficult, tofacitinib may be considered as an alternative therapeutic strategy.

## Introduction

Rheumatoid arthritis (RA) is an autoimmune disorder predominantly characterized by inflammatory synovitis. While RA predominantly affects the joints, it can also Involve skeletal muscles, leading to conditions such as sarcopenia and impaired muscle function. Myositis is a rare extra-articular manifestation of RA. Focal myositis (FM) is a localized inflammatory muscle condition distinct from systemic myositis (1), affecting individual muscles and presenting histologically with features such as inflammatory cell infiltration (macrophages and T cells) and fibrosis.

In this report, we present a case of a long-standing RA patient who developed acute left lumbar region, hip and leg pain, accompanied by elevated C-reactive protein(CRP), creatine kinase(CK), and a palpable subcutaneous mass. Initially misdiagnosed twice as an infection and poorly responsive to antibiotics, the patient was eventually diagnosed with FM, and treatment with glucocorticoids and Janus kinase (JAK) inhibitors yielded excellent results.

## Case presentation

A 58-year-old female with a 10-year history of stable RA, managed with daily leflunomide (20 mg), presented with pain of left lumbar, hip and leg that had persisted for one month and worsened over the past ten days. She reported no recent illness, injury, or travel history and had no family history of myositis. Her RA had been stable, with no notable joint swelling or pain. On physical examination, tenderness was observed in the left iliac and lumbar regions, along with palpable subcutaneous mass and restricted movement of the left lower limb. Laboratory findings revealed elevated inflammatory markers, including CRP (172.6 mg/L), serum amyloid A (>320.0 mg/L), alanine aminotransferase (60 U/L), aspartate aminotransferase (115 U/L), CK (2408 U/L), and lactate dehydrogenase (428 U/L). Tests for antinuclear antibodies, anti-neutrophil cytoplasmic antibody, anti-myositis spectrum antibodies, tuberculosis infection T-cells, and tumor markers were negative.

**Figure 1:**
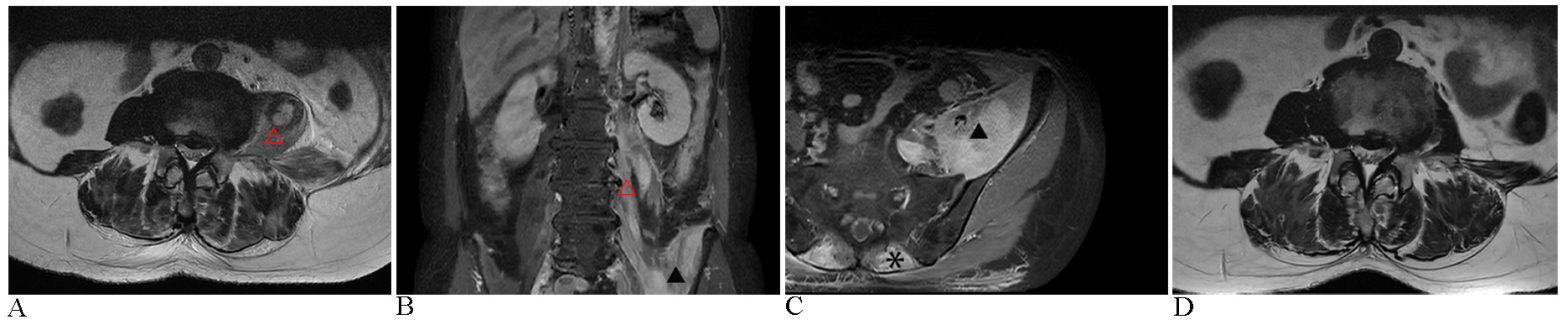
Images before and after the treatment. **(A)** The initial MRI assessment revealed significant swelling of the left psoas muscle (△) on T2-weighted sequences. **(B)** Coronal MRI with contrast enhancement demonstrated heterogeneous high signal intensities in the left iliopsoas (▴) and psoas muscles, following gadopentetate dimeglumine administration. **(C)** Axial MRI with contrast further illustrated diffuse swelling of the left iliopsoas muscles, along with bilateral erector spinae (*), exhibiting a distinct heterogeneous signal intensity; subcutaneous fascial edema was also present in the lumbar region. **(D)** Twenty-five days after initiating continuous glucocorticoid therapy, MRI imaging demonstrated a significant reduction in muscle swelling compared to image A.

**Figure 2:**
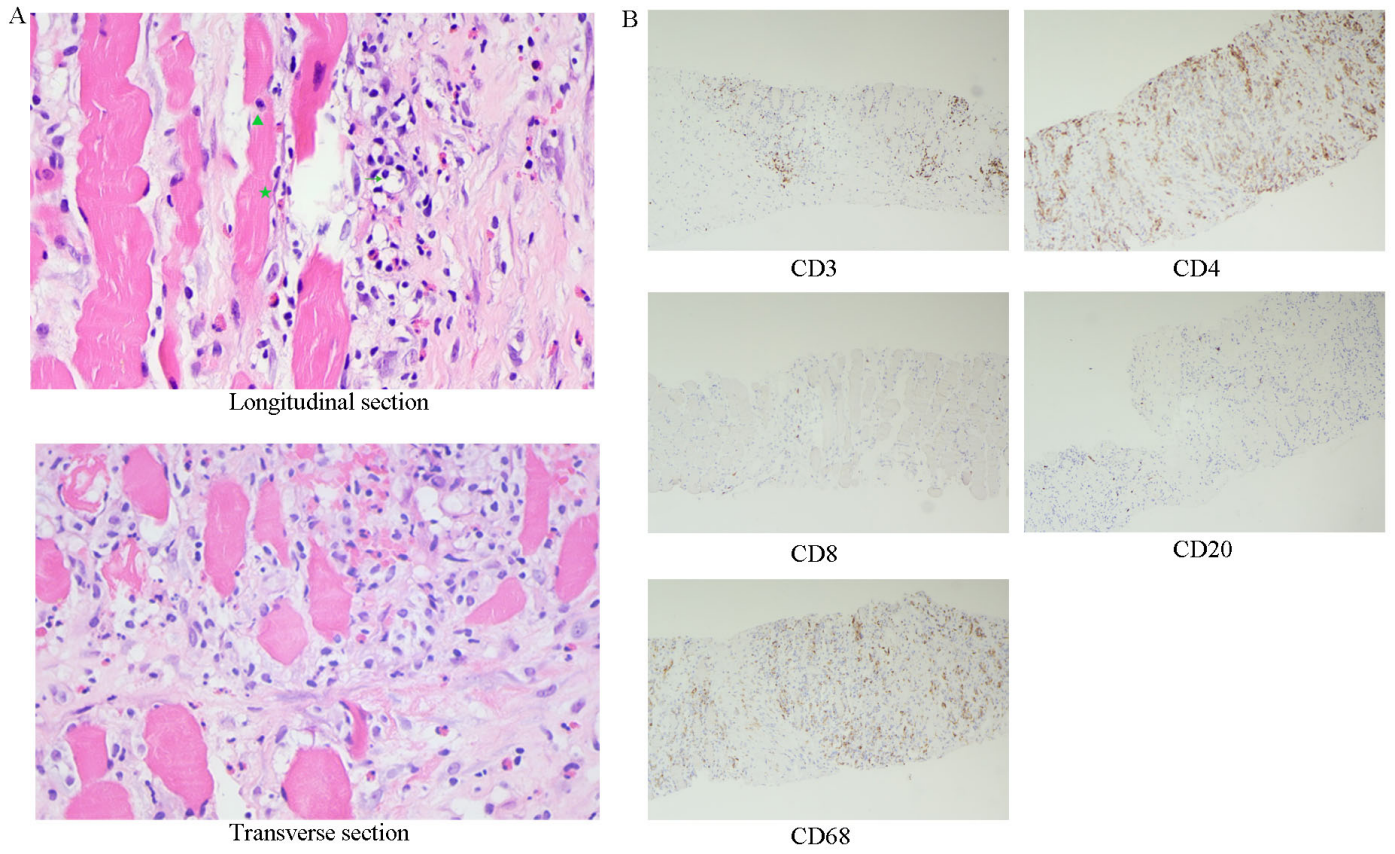
The initial muscle biopsy, along with subsequent immunohistochemical analysis. **(A)** Skeletal muscle tissue exhibits marked fibrous hyperplasia with local proliferation of fibroblasts (★), HE400 x. Additionally, there is notable infiltration of lymphocytes (→), neutrophils (▴), and a minor presence of eosinophils. **(B)** Immunohistochemistry, 100 x.

Magnetic resonance imaging(MRI) showed diffuse swelling of the left iliopsoas, psoas major, and erector spinae muscles, raising suspicion of an infection (**Figure 1**). Ultrasound-guided biopsy revealed striated muscle with localized fibroblast proliferation and inflammatory cell infiltration (**Figure 2**). Initial treatment with levofloxacin and celecoxib was ineffective, and further antibiotic therapy with ceftriaxone and moxifloxacin also failed, accompanied by recurrent low-grade fever.

A rheumatology consultation suggested the possibility of RA-related myositis. Treatment with intravenous methylprednisolone (40 mg daily) led to significant improvement in both pain and fever, as well as reduced inflammatory markers. However, the patient was reluctant to continue with steroid therapy and was transferred to an infectious disease department. Reassessment and muscle biopsy at a different hospital showed collagen fiber proliferation and chronic inflammation in the left erector spinae muscle, but metagenomic sequencing revealed no bacterial infection. Following steroid withdrawal, treatment with cefoperazone-sulbactam and linezolid exacerbated the pain and led to low-grade fever.

The patient asked to return to our hospital for further treatment. Immunohistochemistry of biopsy specimens showed CD3(+), CD4(+), CD8(+ sporadic), CD68(+), and CD20(+ sporadic) cells (**Figure 2**), consistent with FM (2). The patient resumed treatment with methylprednisolone (40 mg daily) and leflunomide, leading to pain relief and normalization of creatine kinase and CRP levels. However, symptoms recurred upon tapering prednisone to 25 mg daily. Tofacitinib (5 mg twice daily) was added to the regimen, resulting in significant symptom improvement after two weeks.

**Figure 3:**
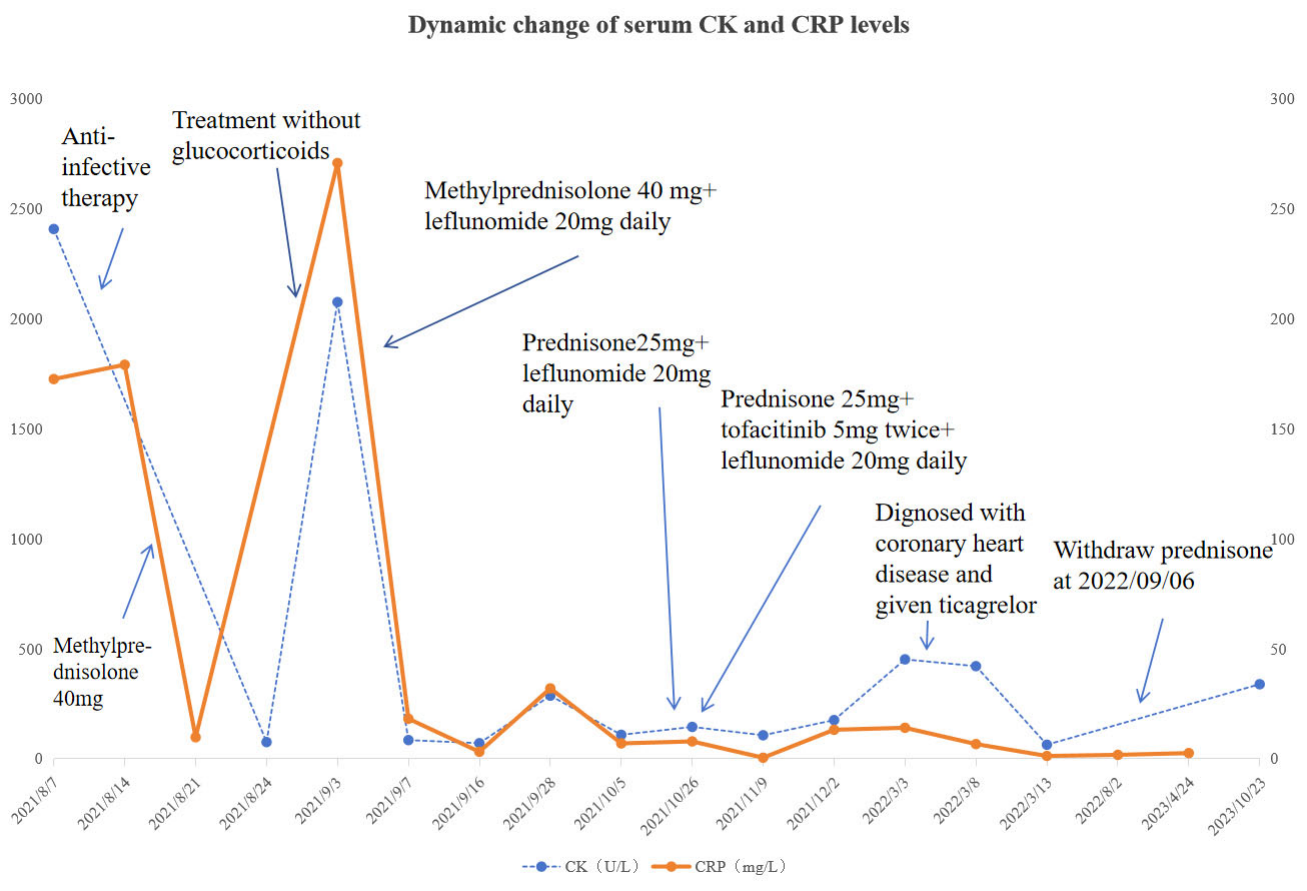
Dynamic change of serum CK and CRP levels during the course. Please see **Supplementary Materials** for more details.

After 11 months, the patient had successfully transitioned from corticosteroids to tofacitinib and leflunomide and maintained a relapse-free status for over two years (**Figure 3**).

## Discussion

FM is a rare idiopathic inflammatory myopathy, with autoimmune disorders as potential etiological factors. Our patient, with a ten-year history of stable RA treated with leflunomide, presented with acute left lumbar region, hip and leg pain, notable muscle inflammation, and elevated muscle enzyme and CRP levels. She was misdiagnosed with infections on two occasions, with inadequate responses to anti-infective therapies, leading to a reconsideration of the diagnosis based on pathological findings. Considering the successful use of tofacitinib in the treatment of polymyositis and vasculitis, we introduced tofacitinib as an innovative therapeutic approach in this case.

Halla JT (3) reported muscle involvement in RA, noting that nearly all RA patients undergoing autopsy exhibited focal lymphocytic and plasma cell infiltration at necrotic muscle sites, termed nodular myositis. However, reports of RA-associated myositis remain scarce. FM is prone to misdiagnosis and must be distinguished from conditions such as polymyositis, infections, and tumors, including soft tissue sarcomas and lymphomas. A previous study (4) documented a case of lymphoma initially misdiagnosed as FM, with the correct diagnosis confirmed after a second biopsy. Additionally, there are instances where FM was radiologically mistaken for rhabdomyosarcoma (5). Our patient experienced elevated CK levels and recurring pain upon steroid tapering, raising concerns about a possible malignancy. Although positron emission tomography/computed tomography was not performed, retrospective analysis indicated that, under immunosuppressive therapy, the patient remained pain-free with normalized CK levels for over two years, allowing us to largely rule out a tumor based on biopsy results. Initially misdiagnosed as infections, the patient underwent anti-infective treatments in both our hospital’s orthopedic department and infection units at other hospitals. However, negative results from multiple blood cultures, muscle biopsies, and metagenomic next-generation sequencing further supported a non-infectious etiology, likely autoimmune in nature, along with the positive response to high-intensity immunosuppression. The patient’s myositis, localized to the iliopsoas, psoas major, and erector spinae muscles, may be more appropriately classified as localized myositis rather than polymyositis. Nonetheless, it is important to note that some cases of FM may evolve into polymyositis (6), warranting long-term follow-up.

The precise etiology of FM in this patient remains unclear. Zarmeena Ali (7) reported a case of RA-associated localized skeletal muscle vasculitis without other manifestations, such as rash or skin ulceration. That patient did not respond to nonsteroidal anti-inflammatory drugs or muscle relaxants but responded well to corticosteroids, with a muscle biopsy revealing non-necrotizing small vessel vasculitis. We surmise that the mechanism underlying the FM in our patient may involve immune-mediated vascular inflammation secondary to RA, leading to thrombosis of the supplying blood vessels, subsequent ischemic necrosis of the localized muscle, inflammatory exudation and muscle fiber regeneration, resulting in a palpable mass. However, both muscle biopsies performed on this patient did not demonstrate any signs of vasculitis. It is important to note that both biopsies were conducted via ultrasound-guided percutaneous biopsy. In contrast, open muscle biopsy may provide greater diagnostic yield, as core needle biopsy may have missed critical tissue features.

Previous studies have suggested that some cases of FM may respond to physical therapy or nonsteroidal anti-inflammatory drugs. However, patients presenting with significant elevations in muscle enzymes often require corticosteroid therapy, especially in cases associated with rheumatic disease-related myositis (8). These patients may experience recurrent episodes, particularly during attempts to taper corticosteroids. In the present case, high-dose corticosteroids combined with leflunomide initially provided rapid symptom relief. However, symptoms recurred when the prednisone dose was reduced to 25 mg daily.

Tofacitinib, an oral JAK inhibitor primarily used in the management of RA, inhibits T-cell proliferation, cytokine secretion, and cytotoxic effects, thereby reducing autoimmune activity. In addition, tofacitinib has demonstrated efficacy in treating vasculitis and polymyositis (9). Given the significant infiltration of macrophages and T cells in FM and the potential for progression to polymyositis or immune-mediated vasculitis in this RA patient, we opted to introduce tofacitinib as an adjunct immunosuppressive therapy. This approach aimed to reduce the patient’s reliance on corticosteroids, and it proved to be highly effective. Recent research (10) also suggests that JAK inhibitors, including tofacitinib, may promote the reprogramming of macrophages towards a more anti-inflammatory phenotype, which could explain its therapeutic success in this case.

## Conclusion

This report presents a case of RA-associated FM successfully managed with tofacitinib. Given the rarity of RA-related FM and the potential for diagnostic challenges, this case underscores the critical importance of early recognition, accurate diagnosis, and timely therapeutic intervention. Tofacitinib may serve as a valuable treatment option for patients struggling with corticosteroid tapering, offering an alternative immunosuppressive strategy to achieve long-term disease control.

## Data Availability

The original contributions presented in the study are included in the article/**Supplementary Material**. Further inquiries can be directed to the corresponding author.
