# Supplementary material for: An effective strategy with tofacitinib for the management of focal myositis with rheumatoid arthritis: a case report
Source: Front Immunol. 2024 Dec 24;15:1502410. doi: 10.3389/fimmu.2024.1502410 (PMC11703955; doi:10.3389/fimmu.2024.1502410)
Supplement: Supplementary file 1 [file DataSheet1.pdf]

Dynamic change of serum CK, ESR and CRP levels during the course

Date	CK（U/L）	CRP（mg/L）	ESR（mm/H）
2021/8/7	2408	172.6	64
2021/8/14		179.1	61
2021/8/21		9.8	51
2021/8/24	76		
2021/9/3	2076	270.8	98
2021/9/7	85	18.2	
2021/9/16	71	3.2	
2021/9/28	287	32	41
2021/10/5	109	7	43
2021/10/26	145	7.9	43
2021/11/9	107	0.5	29
2021/12/2	176	13.2	30
2022/3/3	452	14.1	36
2022/3/8	421	6.7	27
2022/3/13	64	1.3	
2022/8/2		1.8	24
2023/4/24		2.6	27
2023/10/23	339		
